# Extracellular ATP and Toll-Like Receptor 2 Agonists Trigger in Human Monocytes an Activation Program That Favors T Helper 17

**DOI:** 10.1371/journal.pone.0054804

**Published:** 2013-01-31

**Authors:** Christopher Paustian, Patricia Taylor, Terrence Johnson, Min Xu, Nancy Ramirez, Kenneth S. Rosenthal, Suyu Shu, Peter A. Cohen, Brian J. Czerniecki, Gary K. Koski

**Affiliations:** 1 Department of Biological Sciences, Biomedical Sciences Program, Kent State University, Kent, Ohio, United States of America; 2 Department of Integrative Medical Sciences, Northeastern Ohio Medical University, Rootstown, Ohio, United States of America; 3 Harrison Department of Surgical Research, University of Pennsylvania, Philadelphia, Pennsylvania, United States of America; 4 Department of Immunology, Lerner Research Institute, Cleveland Clinic, Cleveland, Ohio, United States of America; University Freiburg, Germany

## Abstract

Strategically-paired Toll-like receptor (TLR) ligands induce a unique dendritic cell (DC) phenotype that polarizes Th1 responses. We therefore investigated pairing single TLR ligands with a non TLR-mediated danger signal to cooperatively induce distinct DC properties from cultured human monocytes. Adenosine triphosphate (ATP) and the TLR2 ligand lipoteichoic acid (LTA) selectively and synergistically induced expression of IL-23 and IL-1β from cultured monocytes as determined by ELISA assays. Flow cytometric analysis revealed that a sizable sub-population of treated cells acquired DC-like properties including activated surface phenotype with trans-well assays showing enhanced migration towards CCR7 ligands. Such activated cells also preferentially deviated, in an IL-23 and IL-1-dependent manner, CD4^pos^ T lymphocyte responses toward the IL-22^hi^, IL-17^hi^/IFN-γ^lo^ Th17 phenotype in standard *in vitro* allogeneic sensitization assays. Although pharmacological activation of either ionotropic or cAMP-dependent pathways acted in synergy with LTA to enhance IL-23, only inhibition of the cAMP-dependent pathway antagonized ATP-enhanced cytokine production. ATP plus atypical lipopolysaccharide from *P. gingivalis* (signaling through TLR2) was slightly superior to *E. coli*-derived LPS (TLR4 ligand) for inducing the high IL-23-secreting DC-like phenotype, but greatly inferior for inducing IL-12 p70 production when paired with IFN-γ, a distinction reflected in activated DCs’ ability to deviate lymphocytes toward Th1. Collectively, our data suggest TLR2 ligands encountered by innate immune cells in an environment with physiologically-relevant levels of extracellular ATP can induce a distinct activation state favoring IL-23- and IL-1β-dependent Th17 type response.

## Introduction

Dendritic cells (DC) are the most potent known antigen-presenting cells and are primarily responsible for sensitizing naïve T cells to antigen. DC activate T cells by supplying antigenic (signal 1) and co-stimulatory (signal 2) signals as well as an additional set of “third signals” that can profoundly affect T cell function [1]. For example, if the cytokine interleukin-12 (IL-12) is present during Th sensitization it is likely that Th1 polarization will occur, resulting in T cells that produce high levels of IFN-γ and correspondingly less (or no) IL-4 and IL-5 [2]. Such cells can be highly effective for dealing with some intracellular parasites [3]. On the other hand, the cytokines IL-23, IL-6, TGF-β and IL-1β have each been implicated by various groups [4]–[9] in promoting the development of IL-17-producing Th17 cells. These Th17 cells appear highly effective against extracellular bacteria, particularly those that colonize mucosal surfaces [10], and have also been implicated in chronic inflammatory pathology associated with some autoimmune diseases [11], [12]. Additional cytokines contribute to the development of other key Th phenotypes including Th2 and T_reg_ [12], [13]. It has therefore become increasingly clear that these individual Th phenotypes represent adaptations for dealing with particular types of infection, or otherwise regulating immune responses. In contrast, dysregulation of these differentiation programs could result in ineffective immune responses against pathogens, debilitating autoimmune pathologies, or perhaps even promotion of carcinogenesis [14].

Such Th-polarizing third signals are produced only conditionally, depending on the precise activation stimuli received by the DC. Many of the signals that activate and otherwise shape DC function belong to the class of so-called molecular “danger signals” [15], which are of two general types. “Exogenous” danger signals are usually microbe-derived, and may also be referred to as “pathogen-associated molecular patterns” (PAMPs), which are absent from normal host tissues but common to broad classes of potential pathogens [16]. These are sensed by a number of specialized transmembrane as well as cytoplasmic receptors, the best studied of which belong to the Toll-like (TLR) family [17], now known to include 10 members in humans. In contrast, “endogenous” danger signals are produced by host cells and released as a consequence of cellular damage or stress [18]. Some endogenous danger signals can also be sensed through TLRs [19], but others activate completely different signaling pathways, for example the sensing of aberrant glycosylation through lectin-like receptors [20].

Interestingly, extracellular adenosine triphosphate (ATP) can be considered both an endogenous and an exogenous danger signal [21]. ATP is normally sequestered at high concentrations in the cytoplasm of cells, with extracellular concentrations remaining characteristically low. Although ATP is usually considered the energy currency of cells, cellular damage can release ATP into extracellular fluids. In addition, it has been recently demonstrated that at least one member of the normal gut bacterial flora (probably *Enterococcus gallinarum*) can secrete large amounts of ATP [22] and that such microbial-derived ATP can influence Th17 development in the intestinal lamina propria [23]. Whether endogenous or exogenous in origin, extracellular ATP can be sensed by DC through receptors of the P2Y and P2X families [24]. This means that ATP could profoundly influence DC function and promote deviation of Th responses toward Th17.

We sought to determine whether the presence of extracellular ATP would affect DC activation and function. Guided by the precedent established for a two signal model for eliciting Th1 polarizing 3^rd^ signals [25], [26], we hypothesized that pairing ATP with an exogenous danger signal, exemplified by select TLR ligands, might likewise induce a unique set of 3^rd^ signals capable of polarizing T cells toward one of the previously-defined Th phenotypes. We found that this indeed was the case. The combination of ATP and, in particular the TLR2 agonists such as lipoteichoic acid LPS from P. gingivalis cooperatively induced human peripheral blood monocytes to acquire many phenotypic and functional features associated with mature DC. These features included activated surface phenotypical markers, tendency to migrate toward CCR7 ligands, and the capacity to selectively promote the activity of Th17 cells.

## Materials and Methods

### Monocyte Culture and DC Activation

Human peripheral blood mononuclear cells were obtained from healthy volunteers via leukapheresis after provision of written informed consent, and in accordance with the principals of the Declaration of Helsinki and NIH guidelines for human subjects, through protocols approved by the Institutional Review Boards of the Cleveland Clinic (08–957) and Kent State University (12–200). Blood products were separated into CD14^pos^ peripheral blood monocyte and lymphocyte-enriched fractions via countercurrent centrifugal elutriation as described previously [27] and cryopreserved. Monocytes were plated at a density of 1.5×10^6^ cells/ml in either 96-, 48- or 24-well tissue culture plates (Costar, Corning, NY) and cultured overnight in macrophage serum-free medium (Life Technologies, Gaithersburg, MD) supplemented with 50 ng/ml recombinant human GM-CSF (Immunex, Seattle, WA), and in some experiments also with 500 U/ml IL-4 (Schering-Plough, Bloomfield, N.J.). Where indicated, cells were pre-treated with 100 uM ATP or GTP control (both from Sigma, St. Louis, MO) 4 hours prior to application of TLR ligand. In some experimental groups, a “standard” DC known to secrete high levels of IL-12 and skew T cell responses toward Th1 [28]–[30] was used for functional comparisons with ATP-treated DC. These were activated with a combination of IFN-γ (100 U/ml, Biogen Cambridge, MA), LPS (*E. coli* K12, 50 ng/ml In VivoGen) and/or R848 (2 ug/ml, a kind gift from 3 M corporation) and are referred to in the text as “DC1”. *Staphylococcus aureus* LTA (10 ug/ml), p[I:C] (50 ug/ml) and flagellin from *B. subtilis* (10 ug/ml) were obtained from In Vivogen. Forskolin (25 uM), dibutyrl cyclic AMP (dbcAMP) (100 uM), Ca ionophore A23187 (50 ng/ml), dideoxyadenosine (50 uM), and cyclosporine A (0.5 ug/ml) were each obtained from Sigma.

### Allogeneic Mixed Leukocyte Cultures

For allosensitization studies, T cells were purified from lymphocyte-rich elutriation fractions using naïve CD45RA^pos^ or total CD4^pos^ T cell isolation columns (R&D, Minneapolis, MN). These were plated in 48-well cluster plates (1×10^6^/well) in RPMI medium supplemented with 5% human AB serum (Cambrex, East Rutherford, NJ). The monocytes previously activated with ATP and/or TLR agonists were harvested 5–6 h after TLR activation and added to T cell cultures at a 1∶10 APC:T cell ratio. For some experiments, cytokine-neutralizing antibodies for IL-12p70 (R&D), IL-12/23p40 (eBioscience, San Diego, CA) and IL-1β (BD Pharmingen, San Diego, CA or R&D) or appropriate isotype controls, were added immediately and one day following co-culture (10 ug/ml). The co-cultures were maintained for 6 days at 37°C and 5% CO_2_. Cells were then harvested, washed and counted prior to re-plating at a density of 1×10^6^ cells/ml on 96 or 48 well tissue culture plates coated with anti-CD3 and anti-CD28 antibody (BD Pharmingen, San Diego, CA) as described previously [31]. Culture supernatants were collected 24 h later and assayed via ELISA for cytokine output. In some experiments, supernatants from 5 day co-cultures were analyzed without subsequent re-stimulation on anti-CD3/CD28-coated plates.

### Cytokine Quantification by ELISA

All culture supernatants were stored frozen at −70C prior to analysis. Sandwich ELISA antibody pairs (with biotinylated secondary antibodies) were used to quantify IL-12p70, IL-12p40, IL-23, IL-6, IL-10, IL-1β, TNF, IL-17 and IFN-γ (BD biosciences, eBioscience, and R&D systems) from 24 h culture supernatants of stimulated MoDCs or allogeneically-sensitized T cells according to manufacturer’s recommendations. Avidin/HRP conjugate (Sigma) and TMB substrate (Kirkegaard and Perry Laboratories, Gaithersburgh MD) were used to develop plates. Color reaction was stopped with 1 N HCL and absorbance read at 450 nm on a Biotek EL×800 microplate reader using Gen5 software with parametric analysis of the standard curve.

### Surface FACS Analysis

FITC- or PE-labeled mAb specific for human CD80, CD86, CD14, CD83, CCR7, and HLA-DR, as well as isotype-matched control mAb were purchased from BD Pharmingen (San Diego, CA). Cells were carefully removed from 48 well tissue culture plates and aliquoted into 5 ml FACS tubes (Falcon) at 0.25–0.5×10^6^ cells/tube. Cells were immediately diluted with azide-containing buffer to inhibit metabolic activity. They were then washed and Fc blocked with human IgG (Sigma, St. Louis, MO) for 10 minutes. Then cells were stained with labeled Abs at concentrations previously optimized for each stain. After at least an hour of staining at 4°C, cells were washed thoroughly and resuspended in 0.5 ml 4% paraformaldehyde (PFA) before analysis on a FACSCalibur flow cytometer (Beckton Dickinson, San Jose, CA) running CellQuest analysis software. A gate was defined in all FACS analysis based on size and granularity to exclude cellular debris.

### Intracellular FACS Staining

FITC- and PE-labeled mAb specific for IL-12p70, IL-12p40, HLA-DR and isotype matched controls were purchased from BD Pharmingen. Cells were treated with 10 ug/ml Brefeldin A (Sigma, St. Louis, MO) 4–5 hours after TLR stimulation to inhibit cytokine secretion. Otherwise, culture, harvest and extracellular staining were performed as described above. After washing off excess HLA-DR surface stain, cells were fixed and permeabilized with BD Pharmingen Cytofix/Cytoperm buffer for at least one hour at 4°C. Cells were then washed with BDPharmingen Permwash, which was used as a diluent for subsequent steps. Next, cells were stained with fluorescently-labeled Abs for at least 2 hours at 4°C. Cells were then washed and resuspended in 4% paraformaldehyde, and analysis carried out as described above.

### In vitro Chemotaxis Assay

Monocytes were cultured as described above. After overnight culture, the cells were treated with ATP (100 ng/ml) or IFN-γ (1000 U). Four hours later LTA (10 ug/ml), LPS (50 ng/ml) or R848 (2 ug/ml) were added to respective groups. Cells were harvested three hours later and counted by trypan blue dye exclusion and resuspended in SFM with 50 ng/ml GM-CSF at a final concentration of 1×10^6^ cells/ml. A total of 600 µl/well SFM supplemented with CCL19 (250 ng/ml) and CCL21 (250 ng/ml) (R&D Systems, Minneapolis-St. Paul, MN) was added to a 24-well plate. Then, 5.0-µM pore diameter transwell inserts (Corning, Corning NY) were placed in the wells and 2×10^5^ cells (200 ul) were added into the transwell inserts. After incubation for 3 h, the transwell inserts were removed and cells in the lower chamber were recovered in equal volumes and subsequently counted by flow cytometry for one minute. Specific migration rate represents the number of cells counted in wells containing CCL19+ CCL21 minus the number migrating into wells containing no chemokines.

### Immunoblotting

Freshly-harvested cells were washed twice with ice-cold phosphate-buffered saline and extracted in Cell Extraction Buffer (Biosource International, Camarillo, CA) supplemented with a protease inhibitor cocktail tablet (Roche Indianapolis, IN). Samples were clarified, and boiled for five minutes in the presence of dithiothreitol (Molecular Probes, Eugene, OR). Proteins were then separated on a 4–16% bis-tris gradient gel and electrotranfered onto PVDF membranes (both from Invitrogen, Carlsbad, CA). The membranes were probed for p19 or GAPDH with goat anti-human primary antibodies followed by HRP-conjugated bovine anti-goat secondary antibodies (Santa Cruz Biotechnology, Santa Cruz, CA). Blots were visualized by enhanced chemiluminescence and recorded on Amersham Hyperfilm (both from Amersham Biosciences UK, Buckinghamshire, UK).

### Statistical Analysis

Statistical significance between groups was determined using a paired Student’s T test, with p values of 0.05 or less considered significant.

## Results

### ATP and LTA Act Synergistically on Monocytes to Induce Select Inflammatory Cytokines

**Figure 1:**
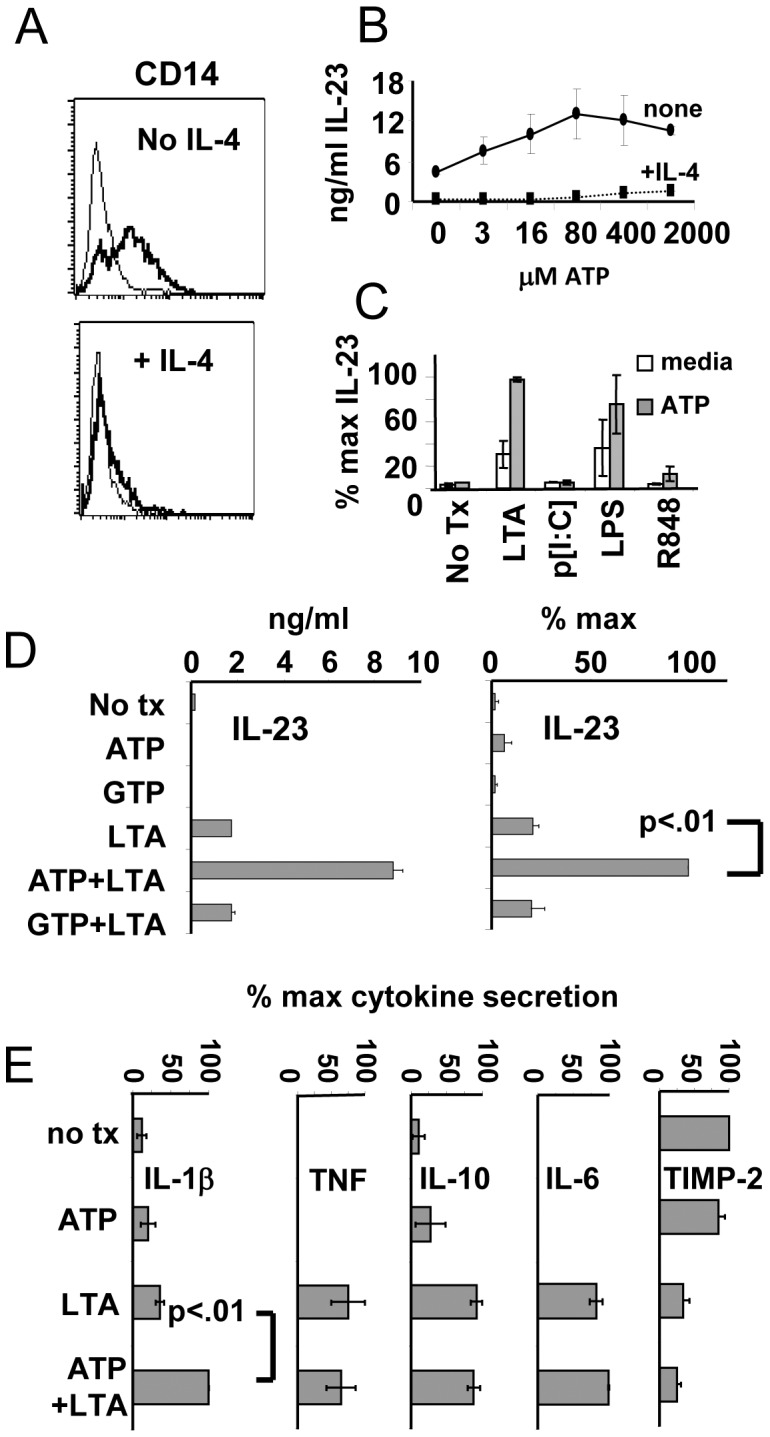
LTA and ATP synergistically induce a unique and restricted cytokine secretion profile in monocytes. (A) FACS analysis of monocytes stained for CD14 24 hours after culture in GM-CSF (top panel) or GM-CSF+IL-4 (bottom panel). Panels representative of 3 experiments with different donors. (B) IL-23 ELISA analysis from cells cultured in SFM supplemented with GM-CSF or GMCSF+IL-4 overnight, exposed to ATP for 4–6 h at indicated concentrations, then matured with S. aureus LTA (10 ug/ml) for an additional 24 h. Results from one donor representative of 3 experiments with different donors. (C) IL-23 ELISA analysis of 24 h culture supernatants from GM-CSF-cultured monocytes pre-treated with ATP followed by application of a set of different TLR agonists. LTA: lipoteichoic acid of S. aureus (TLR2); LPS: lipopolysaccharide from K12 strain of *E. coli* (TLR4); p[I:C]: polyinosinic:polycytidylic acid (TLR3), and R848 (TLR8). Results shown +/− standard deviation for 2 donors. (D) IL-23 production by monocytes pre-treated with ATP or GTP (100 uM) followed by application of LTA. Left panel: results from individual donor. Right panel: mean percent maximal values from 6 individual donors. (E) Effects of ATP and LTA on the secretion of additional cytokines measured in culture supernatants 24 h post-activation. Data from panels C and D represent combined results from 6 donors in three separate experiments +/−SEM.

Based on the 2-signal model for inducing optimal Th-polarizing activity from DCs [25], [26], we hypothesized that the combination of ATP and exogenous danger signals (in the form of Toll-like receptor ligands) would act synergistically to induce a distinct pro-inflammatory APC phenotype. Since we suspected IL-23 would play an important role for this phenotype, we wanted to test this activating combination both on iDC as well as undifferentiated CD14^pos^ monocytes. This distinction was made because the differentiation of monocytes into iDC usually requires pre-exposure to IL-4, and IL-4 has been shown to strongly suppress IL-23[31]. We therefore began by comparing overnight cultures of monocytes maintained in SFM with GM-CSF only (cells that retain CD14^pos^ monocyte/macrophage character) with cells cultured in both GM-CSF and IL-4 (that become CD14^neg^ iDC) (Figure 1A). When we treated overnight cell cultures with varying concentrations of ATP, then activated them with *S. aureus* LTA, it was clear that the levels of IL-23 produced were much higher for the monocytes than iDC (Figure 1B). We next examined, with CD14^pos^ monocytes, the IL-23-inducing capacity of additional TLR ligands (*S. aureus* LTA;TLR2, p[I:C];TLR3, *E. coli* LPS;TLR4, R848; TLR7/8) with or without added ATP. We initially maintained our focus on IL-23 because this cytokine is regarded as critical for the support of Th17-dominated immunity. Interestingly, we found that of the tested TLR ligands, only LTA and LPS were capable of strongly synergizing with ATP for the production of IL-23 (Figure 1C). We therefore selected LTA for detailed study, and conducted all further experiments directly on monocytes cultured in the absence of IL-4 (unless otherwise indicated).

We next sought to establish the specificity of ATP enhancement of IL-23 production by comparing it with another nucleotide triphosphate, GTP, across a larger cohort of healthy donors. Monocytes from overnight cultures were pre-treated with nucleotide triphosphates (100 uM) for 4–6 h followed by application of LTA (10 ug/ml). After 24 h of further incubation, culture supernatants were collected and evaluated by ELISA. Figure 1D (left panel) displays the response of the monocytes from a representative healthy donor. Cells left untreated, or treated with ATP or GTP only produced low or undetectable levels of IL-23. Cells treated with LTA alone produced low to moderate levels of IL-23. However, cells treated with both ATP and LTA produced much higher levels of IL-23. GTP, however, did not display this synergy when combined with LTA indicating that this effect was nucleotide-specific. The generalized nature of this response was born out in experiments with 6 donors where ATP plus LTA treatments posted statistically significant enhancement of IL-23 production (ranging from 2–10 fold) compared to LTA stimulation alone (Figure 1D, right panel). We next expanded the battery of evaluated cytokines to include several others associated with inflammation, including IL-1β, IL-6, IL-10 and TNF (Figure 1E). IL-1β secretion significantly increased in a manner similar to IL-23, while IL-6, IL-10 and TNF showed little or no change. Tissue inhibitor of matrix metaloproteinases 2 (TIMP-2) secretion, already suppressed by LTA alone, appeared further inhibited by the addition of ATP for most donors (n = 6), but this further decrease was not statistically significant. It should be noted that in addition to ELISA, we screened supernatants using an antibody cytokine array that assessed the relative levels of 64 inflammation-associated cytokines. The arrays confirmed the up-regulation of IL-1β and suppression of TIMP-2, but uncovered no evidence of other products whose expression was modulated by combined LTA and ATP treatment (data not shown). This suggests that combined LTA and ATP treatment activated a highly selective cytokine secretion program.

### ATP and LTA Act Synergistically to Induce the Surface Expression of Activated DC Markers

**Figure 2:**
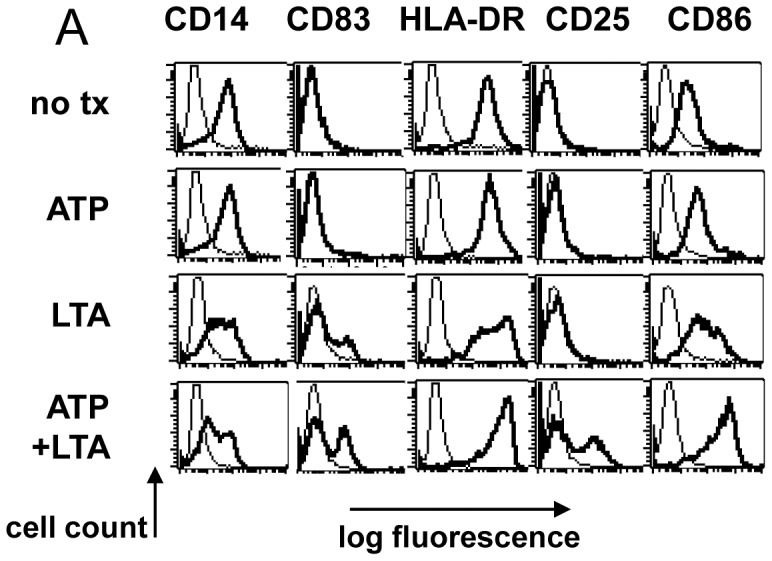
LTA and ATP act synergistically to induce elements of mature DC phenotype from human monocytes. A. FACS analysis of monocytes 24 hours after LTA-induced maturation. Harvested cells were stained with PE- or FITC-conjugated antibodies of the indicated specificities (heavy traces) or isotype-controls (light traces). Results shown from one of four representative donors.

We next determined whether combined ATP and LTA treatment affected the expression of a set of surface markers associated with DC activation (Figure 2). Cells cultured in GM-CSF retained monocytic features including sustained expression of CD14, absence of the DC activation markers CD83 and CD25, and low to moderate levels of HLA-DR and CD86. As expected, treatment with LTA resulted in decreased CD14 expression and up-regulation of CD83 (but not CD25) in a sub-population of cells, as well as increased expression of HLA-DR and CD86. For cells treated with both ATP and LTA, however, CD14 was maximally down-regulated, while levels of CD83 were clearly enhanced more than for cells receiving only LTA. Furthermore, a distinct population of CD25-expressing cells was notable, where none were detectable with single-agent treatment. HLA-DR and CD86 were also uniformly up-regulated as compared to LTA or ATP alone. Therefore, the combined treatment acted synergistically to promote the surface immunophenotype often associated with mature Mo-DC in a sizable fraction of monocytes.

### ATP+LTA Act Synergistically to Induce Chemotaxis

**Figure 3:**
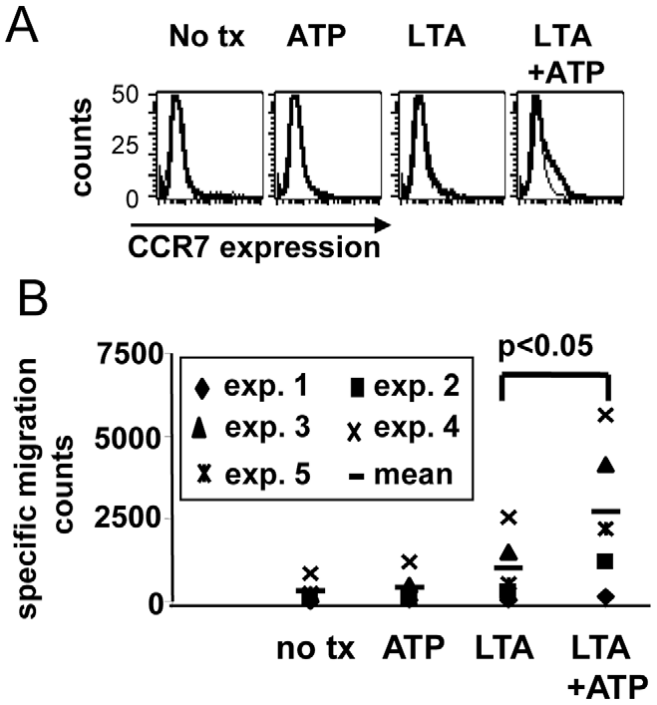
LTA and ATP synergistically promote migration toward CCR7 ligands. (A) FACS analysis of activated monocytes stained with FITC-anti CCR7 antibody. Results shown from one of 4 representative experiments with different donors. (B) Harvested cells were placed in the top chambers of transmigration cluster plates suspended over a lower chamber containing SFM supplemented with 250 ng/ml each CCL19 and CCL21. After 1.5 h all cells from lower chambers were harvested in equal volumes and counted for 60 seconds using flow cytometry. Results shown include 5 separate experiments with different donors.

We next turned our attention to the evaluation of the functional properties of these cells. An important functional trait characteristic of activated DCs is the tendency to migrate toward soluble factors facilitating lymph node homing, including CCL19 and CCL21. We first examined by FACS analysis whether DC preparations expressed the appropriate chemokine receptor, CCR7 (Figure 3A). FACS analysis showed that neither untreated monocytes, nor cells treated with ATP or LTA alone expressed any detectable CCR7. Combined treatment with ATP plus LTA, however, induced detectable CCR7 expression in a subpopulation of the cells. These cell preparations were therefore tested in a transwell migration assay to determine whether migratory capacity was also enhanced by combined ATP and LTA treatment (Figure 3B). We found that the combination of ATP plus LTA induced significantly greater migratory behavior (p<.05) toward a CCL19+CCL21 gradient than LTA alone, demonstrating cooperative enhancement of another key functional property associated with activated DCs.

### ATP+LTA Treated Monocytes Induce a Th17 Cytokine Profile from Allogeneic CD4^pos^ T cells through an IL-23- and IL-1β-dependent Mechanism

**Figure 4:**
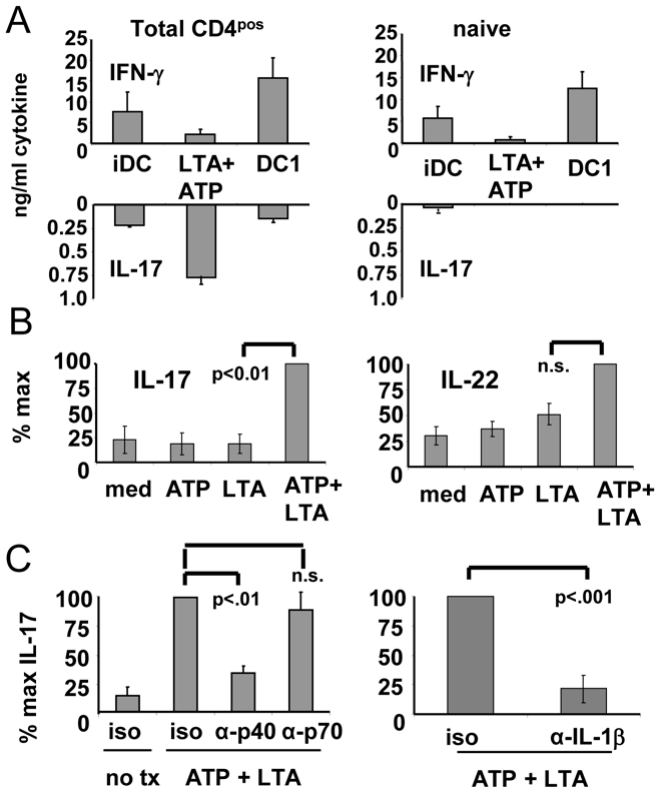
LTA and ATP act synergistically to promote IL-23 and IL-1β dependent Th17-activation. Overnight cultures of monocytes were treated with ATP (100 uM) for 4–6 hrs followed by application of 10 ug/ml LTA. Other groups received either no treatment (iDC) or a standard DC1 polarizing regimen (see Materials and Methods). Cells were harvested 5–6 h after TLR ligand application to serve as stimulators. (A) Monocytes were co-cultured with purified CD4^pos^ (naïve+memory) or CD4^pos^/CD45RO^neg^ (naïve) T cells at 1∶10 stimulator:responder ratios. After 6 days co-culture, T cells were harvested and re-cultured on tissue culture plates pre-coated with anti-CD3 and anti-CD28 antibodies, with supernatants harvested 24 h later and analyzed by ELISA for IFN-γ and IL-17. Results shown from 6 allogeneic APC:T cell pairings +/− SEM. (B) Culture supernatants removed from initial co-cultures of total CD4^pos^ populations on day 5 (prior to cell harvest and antibody re-stimulation) were analyzed by ELISA for IL-17 and IL-22. Results for (B) expressed as mean percent maximums from 6 allogeneic APC:T cell pairings +/− SEM. (C) Co-cultures of APCs and total CD4^pos^ T cells were initiated in the presence of IL-23/12 (p40), IL-12 (p70) or IL1β –neutralizing MoAb, or isotype controls, with culture supernatants harvested on day 5 and analyzed for IL-17 production by ELISA Results from 3 allogeneic APC:T cell pairings +/− SEM.

Because of the cytokine secretion profile of the cells treated with ATP plus LTA, we suspected that these cells would be particularly efficient at polarizing Th cells toward the Th17 phenotype. We therefore purified populations of total CD4^pos^ T cells as well as the CD45RO^neg^ (naïve) subpopulation and co-cultured these cells with allogeneic stimulators in the form of iDC, polarized DC1 (expected to induce polarized Th1 responses) [32] or the ATP+LTA-treated cells. After a week of in vitro activation and expansion, T cells were polyclonally re-stimulated, and culture supernatants evaluated for the presence of the archetypical Th1 cytokine IFN-γ or the defining Th17 cytokine, IL-17. For unfractionated CD4^pos^ cells (containing both naïve and memory populations), it was clear that iDC had no pronounced Th polarizing capacity, sensitizing T cells for relatively low levels of both IFN-γ and IL-17 upon subsequent re-stimulation (Fig 4A). On the other hand, highly polarized DC1 sensitized Th cells for strong IFN-γ responses, with only low levels of IL-17. In contrast, the stimulators activated with combined ATP and LTA induced in Th very high levels of IL-17 with almost no IFN-γ. It appeared, however, that the Th17-skewing effect of these cells was restricted to the memory population, since when memory cells were removed from the CD4^pos^ population (leaving only naïve cells), IL-17 could not be induced by any of the DC preparations (right panel). When analyzing a larger cohort of allogeneic donor pairings (n = 6) for functional synergy induced by ATP+LTA (Figure 4B), IL-17 expression was found to significantly increase (left panel) when compared to the Th17-activating capacity of single agent-treated stimulators. The Th17-associated cytokine IL-22 was also observed to display a trend for synergistic enhancement for all donor pairings (right panel), though the actual degree of increase failed to meet the criteria of significance by the Student’s t-test.

Evidence has accumulated that soluble factors secreted by DCs (third signals) play an important role in determining the phenotype of sensitized T cells. For example, IL-12 p70 promotes Th1 development [2]. Because both IL-23 and IL-1β were enhanced by combined ATP/LTA treatment, we were particularly interested in determining whether either of these two DC-produced cytokines contributed to the pronounced deviation toward the IL-17-secreting phenotype we observed in the memory-containing Th fraction. To address this issue, we performed neutralization experiments using a monoclonal antibody directed against the p40 heavy chain shared by both IL-23 and IL-12 (and capable of neutralizing both cytokines). This exerted a strong suppressive influence (p<.01) on IL-17 production when added to sensitizing DC: CD4^pos^ co-cultures (Figure 4C, left panel). On the other hand, a IL-12 p70-specific antibody did not block IL-17 production, strongly suggesting that IL-23, but not IL-12, was responsible for favoring IL-17 production by Th cells. We also found that IL-1β neutralizing antibodies, but not isotype controls, likewise suppressed (p<.001) IL-17 production (right panel). In contrast, IL-6-neutralizing antibodies showed no effect (data not shown). These studies strongly suggest that both IL-23 and IL-1β contribute to Th17 activation induced by the DC-like cells treated with ATP plus LTA.

### ATP Enhances IL-23 Secretion via Increased p19 Production

**Figure 5:**
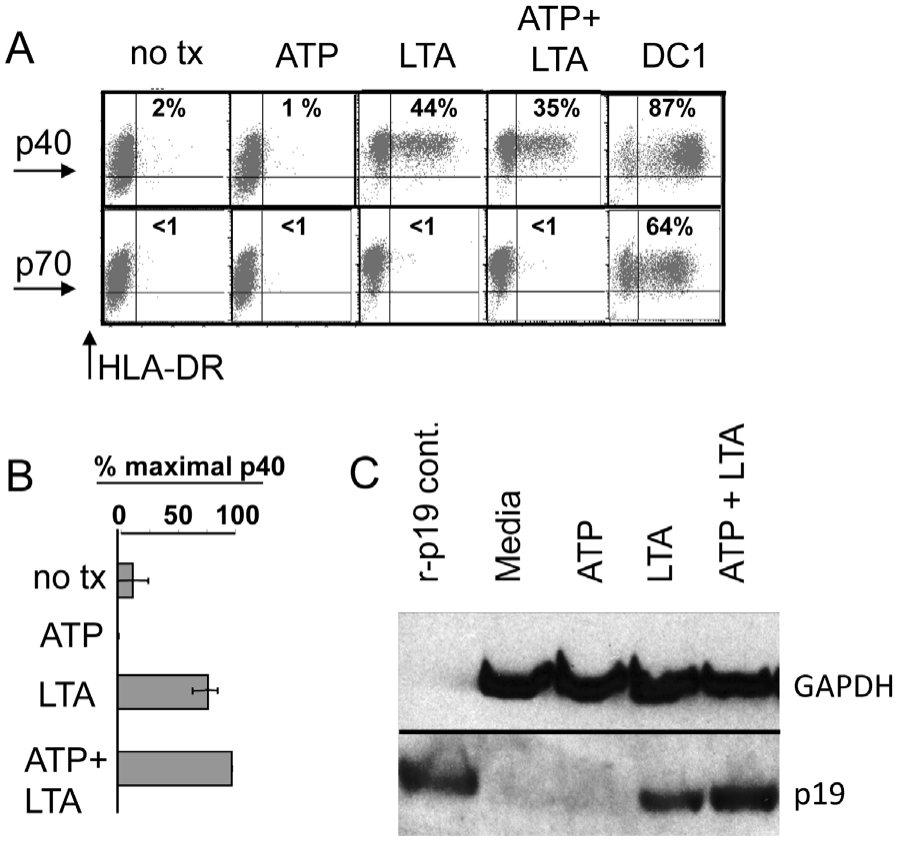
Synergistic up-regulation of IL-23 is regulated through production of p19 but not the p40 subunit. (A) Monocytes pre-treated with ATP were stimulated with LTA for 4 h prior to addition of Brefeldin A. After 24 h further incubation, harvested cells were surface-stained with FITC-labeled anti-HLA-DR, permeablized and intracellular-stained with PE-labeled anti- IL-12 p70 or anti IL-12/23 p40 and subjected to FACS analysis. Percentiles in each panel denote proportion of double-positive cells. (B) Duplicate culture supernatants from cells not receiving Brefeldin A were analyzed for IL-12/23 p40 secretion by ELISA. Results shown are mean percent maximum values from 4 donors +/− SEM. (C) Western blot analysis of whole cell extracts probed with anti-IL-23 p19 antibody, or GAPDH as loading control. Results representative of 2 donors.

Because of the demonstrated important role of IL-23 as a third signal for sustaining Th17 activity, we examined the protein-level regulation of IL-23 induced by combined treatment with ATP and LTA. Because IL-23 is a heterodimeric cytokine composed of a p40 heavy chain (shared with IL-12) and a p19 light chain, regulation of IL-23 production could be achieved through transcriptional/translational control of either of these subunits (or perhaps both). We therefore sought to measure the production of each subunit individually at the protein level. We first used intracellular FACS staining using a p40-specific antibody (Figure 5A). We showed that there were <1% p40-staining cells both in untreated monocytes and cells treated with ATP only. Cells treated with LTA contained about 44% p40^pos^ cells. The addition of ATP to LTA did not appear to increase the percentage of p40-staining cells or the staining intensity. In contrast, polarized DC1 cells were almost uniformly positive for p40 expression. As expected, only the polarized DC1 expressed IL-12 p70. We also examined the secretion of p40 by ELISA (Figure 5B). As with the intracellular FACS, ELISA detected no enhanced p40 production to account for observed increases in IL-23. We next examined the production of the p19 subunit. Because neither p19-specific FACS nor ELISA reagents are commercially available, we used Western blot analysis to look for changes in p19 expression (Figure 5C). We found detectable enhancement of the levels of p19 with co-treatment using ATP and LTA. This suggests that cooperative up-regulation of IL-23 induced by ATP plus LTA is achieved through control of p19, rather than p40 expression.

### ATP Enhances LTA Induced IL-23 Secretion via an Adenylate Cyclase-dependent but not an Ionotropic Pathway

**Figure 6:**
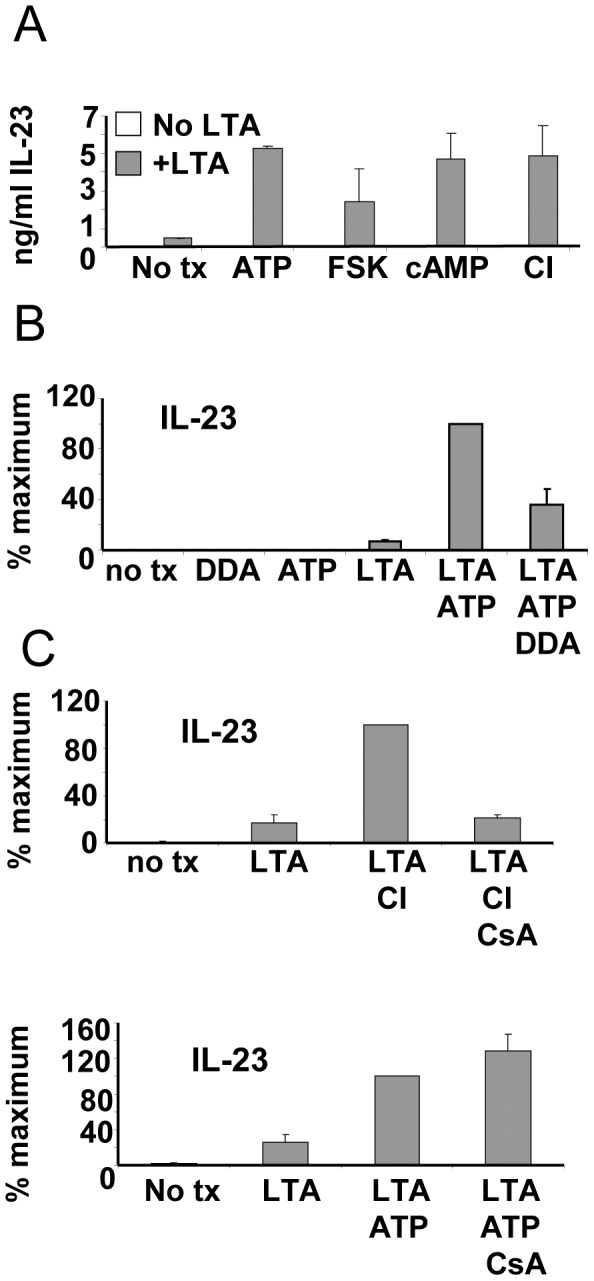
ATP enhances IL-23 production through an adenylate cyclase-dependent but not an ionotropic pathway. (A) Overnight cultures of monocytes were pre-treated with ATP, Forskolin (FSK), dibutyrl cyclic AMP (dbcAMP) or calcium ionophore A23187 (CI) prior to application of LTA. Culture supernatants were harvested 24 h later and analyzed by ELISA for IL-23. (B) Monocytes were treated for 30 min with the adenylate cyclase inhibitor dideoxyadenosine (DDA) prior to 4h ATP treatment. Cells were then activated with LTA and 24 h supernatants analyzed by ELISA for IL-23. (C) Monocytes were pre-treated with calcineurin-dependent phosphatase inhibitor, cyclosporine A (CsA) for 30 min prior to 4 h treatment with CI (upper panel) or ATP (lower panel). Cells were then activated with LTA. After 24 h culture supernatants were removed and analyzed for IL-23 by ELISA. Data from all panels derived from 3 separate experiments with different donors +/− SEM.

We next sought to determine the mechanism by which ATP affects IL-23 expression. Extracellular ATP is sensed through purinergic receptors belonging to either the P2Y or the P2X family [33]. P2Y family members are G-protein coupled receptors, some of which activate adenylate cyclase and produce cAMP, which in turn activates additional downstream signaling pathways. P2X family members are ionotropic, acting as ion channels for Ca^++^, K^+^ and other charged species whose intracellular concentrations can also affect discrete signaling pathways. We therefore began this series of studies by examining whether pharmacological agonists of either the cAMP pathway or various ionophores could enhance IL-23 production elicited by LTA in a manner similar to that achieved by ATP (Figure 6A). As before, LTA as a single agent induced relatively low levels of IL-23 that were greatly enhanced when combined with ATP. Very similar increases in IL-23 secretion were observed when ATP was substituted with the adenylate cyclase agonist, forskolin. The same effects were observed when the non-degradable cAMP analog, dibutyryl cAMP was employed. These mutually-supporting results pointed strongly to the P2Y-adenylate cyclase-cAMP axis for the control of IL-23 production. We were therefore surprised to find that calcium ionophore A23187 also displayed nearly identical synergy with LTA for enhancing IL-23 production (potassium ionophore nigericin had no effect - data not shown). This meant that either ATP enhanced IL-23 production via both signaling pathways simultaneously, or that we had serendipitously identified a second IL-23-amplifying pathway that was unrelated to ATP. To resolve this issue, we turned to the use of pathway antagonists, and attempted to block the IL-23 enhancement induced by ATP. We started by using the adenylate cyclase antagonist, 2′- 5′dideoxy adenosine (Figure 6B). This agent clearly exerted strong inhibitory action on ATP-enhanced IL-23 production, confirming that ATP functions through an adenylate cylcase-dependent mechanism. To examine whether calcium signaling contributes to ATP-enhanced IL-23 production, we utilized cyclosporine A (CsA), which antagonizes the major calcium-signaling axis through inhibition of the calcium-calmodulin-dependent protein phosphatase, calcineurin. We found that pre-treatment with CsA almost totally blocked the capacity of calcium ionophore to enhance LTA-induced IL-23 production (Figure 6C). However, CsA had no inhibitory effects on IL-23 production enhanced by ATP (Figure 6D). These experiments therefore appeared to indicate that ATP acts through an adenylate cyclase-dependent mechanism to enhance IL-23 production rather than a calcium-dependent one, suggesting that P2Y rather than P2X receptors are involved. However, we also uncovered a novel calcium-dependent pathway that enhances IL-23 production through a mechanism distinct from that utilized by ATP. Although the mechanism by which CI enhances IL-23 production remains undetermined, we have demonstrated previously that calcium signaling enhances NF-κB activation [34], and recent evidence points to a possible role for NF-κB in regulating IL-23 p19 [35], offering a plausible explanation for this observation.

### Different LPS Preparations that Signaling Either through TLR2 or TLR4 Appear to Differentially Induce IL-12 Family Members that Regulate Induced T cell Phenotype

**Figure 7:**
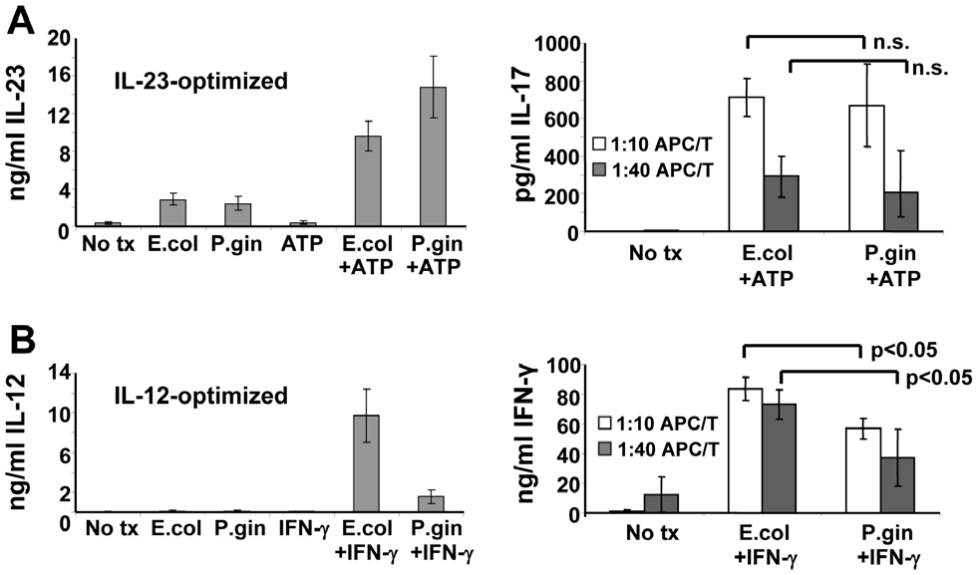
Differential regulation of IL-12 and IL-23 by structurally-divergent lipopolysaccharides leads to distinct Th-deviating profiles. (A) Monocytes cultured in SFM supplemented with GM-CSF only (IL-23 optimized). Cells were pre-treated with ATP for 4 h prior to application of *E. coli* K12 or *P. gingivalis* LPS. Culture supernatants were harvested 24h later and analyzed for IL-23 by ELISA (left panel). Monocytes from replicate cultures were harvested 6 h after LPS activation and co-cultured with CD4^pos^ Th at indicated stimulator:responder ratios. Co-culture supernatants were harvested 5 days later and analyzed for IL-17 production (right panel). (B) MoDC cultured overnight in SFM with GM-CSF plus IL-4 (IL-12 optimized). Cells were then pre-treated with IFN-γ (IL-12 optimized) for 4–6 h prior to application of 500ng/ml LPS from either *E. coli* K12 strain or *P. gingivalis*. Culture supernatants were harvested 24 h later and analyzed for IL-12 p70 by ELISA (left panel). MoDC harvested from replicate cultures were were co-cultured with allogeneic CD4^pos^ T cells at indicated stimulator:responder ratios with supernatants harvested 5 days later and assayed for IFN-γ production by ELISA (right panel). Data from 4 separate allogeneic APC”T cell pairings +/− SEM.

Finally, we sought to determine whether signaling through TLR2 versus TLR4 predisposed DCs toward functional profiles that favored either Th1 or Th17. We undertook this line of investigation because our initial studies showed that the TLR2 agonist, *S. aureus* LTA efficiently synergized with ATP to induce IL-23 in the absence of IL-12, while the TLR4 agonist, *E. coli* LPS, showed somewhat weaker activity. On the other hand, we and others have demonstrated previously that *E. coli* LPS, under the right circumstances (for example, in conjunction with IFN-γ), can powerfully induce polarized DC1 activity with associated high-level secretion of IL-12 p70 [26], [28]. In other studies we have used a special clinical-grade of LPS to drive DC1 polarization for the production of experimental vaccines to treat early breast cancer [29]. In order to make the most meaningful comparison of a TLR2 to a TLR4 agonist, we chose to test standard E. coli K12 LPS against LPS from P. gingivalis (the etiological agent of periodontal disease). Although highly similar molecules, several structural differences make E. coli LPS a TLR4 ligand while P. gingivalis LPS is a ligand for TLR2 [36]. These two molecules, though structurally similar, should reveal functional differences in DCs activated with TLR2 versus TLR4 ligands. We therefore stimulated cultured monocytes with either *E. coli* or *P. gingivalis* LPS under conditions specifically optimized for either maximized IL-23 production in the absence of IL-12 (i.e. in conjunction with ATP), or under circumstances that favor high-level IL-12 production (i.e. in conjunction with IFN-γ). We found that LPS from *E. coli* and *P. gingivalis* each induced comparable levels of IL-23 as single agents at the tested dose of 500 ng/ml used throughout this experimental series. When paired with ATP, levels of IL-23 induced by both types of LPS were greatly enhanced although the effect was more pronounced with *P. gingivalis* LPS (Figure 7A left panel). IL-12 was not detected in supernatants from any of these treatment groups (data not shown). The relatively small differences in IL-23 production between E. coli and P gingivalis LPS-activated cells, however, were not sufficient to differentially enhance IL-17 production when these cells were used as stimulators for allogeneic CD4^pos^ T cells (Figure 7A, right panel). On the other hand, when tested under DC1-polarizing conditions, *E. coli* LPS was a potent inducer of IL-12 in the presence of IFN-γ (as expected), while *P. gingivalis* LPS, under otherwise identical conditions, proved very poor by comparison (Figure 7B left panel). These differences were reflected in the activated DC’s capacity to skew allogeneic Th responses toward the Th1 phenotype, with *E. coli* LPS-activated APC inducing significantly more IFN-γ from allosensitized T cells than those activated with *P gingivalis* LPS (Figure 7B right panel). These studies suggest the possibility that distinct types of LPS from different bacterial organisms that signal through discrete TLRs can exert significantly different functional influences on elements of innate immunity, which in turn might influence adaptive immunity.

## Discussion

Earlier studies by others showed that ATP modulates levels of IL-1β and IL-23 produced by MoDC [37], [38]. Since these two cytokines are known to promote Th17 development [9], [39], we were particularly interested in determining whether such ATP-enhanced capacity for cytokine production would suffice to deviate immune responses toward this Th phenotype. We showed that MoDC derived after culture in IL-4-containing medium, produced only slight amounts of IL-23 when treated with certain TLR ligands, with ATP only very modestly enhancing this effect (Figure 1). In contrast, monocytes that were not pre-differentiated into iDC by the inclusion of IL-4, (as evidenced by sustained expression of CD14) produced much higher levels of TLR-induced IL-23 when activated by select TLR ligands. These IL-23 levels were amplified several-fold in the presence of ATP, with IL-1β production being likewise enhanced. These results suggest that the less-differentiated CD14^pos^ monocytes are far more susceptible to ATP-containing danger signal combinations than cells that have already fully committed to the DC differentiation program. These findings are therefore in agreement with other studies demonstrating that monocytes are, or can become, highly efficient stimulators of Th17 cells, even in comparison with DC [8], [40], and that IL-4 can suppress IL-23 production [31]. Interestingly, paired LTA and ATP signals also acted synergistically to induce a battery of other DC-associated characteristics including chemotaxis toward CCR7 ligands and an activated DC surface phenotype in a sizable population of treated cells.

The paired combination of select TLR ligands plus ATP induced a highly specific enhancement of cytokines among those tested, particularly IL-23 and IL-1β. Both cytokines appeared to be necessary for maximal Th17-promoting activity of the DC in allogeneic MLR studies. But what are the likely mechanisms driving enhanced production of these cytokines so critical for supporting Th17? The most probable mechanisms include activation of the inflammasome. At the core of the inflammasome is a molecular pattern recognition receptor of the nucleotide-binding domain leucine-rich repeat-containing receptor (NLR) family. Collectively these NLRs are capable of initiating inflammatory responses against a broad range of stimuli [41]. One of the most studied NLR family members is NLRP3 (also known as NALP3 and cryopyrin). Unlike TLRs, NLRs are cytoplasmically-localized and respond to molecular danger signals localized to the cytoplasm [42]. Upon activation, the NLR forms a multimolecular inflammasome in conjunction with an adaptor protein known as ASC (apoptosis-associated speck-like protein containing a CARD) and activated caspase-1, which acts on the pro form of IL-1β, transforming it into the biologically-active form [43], [44]. Interestingly, it has been demonstrated by others that TLR signaling can promote the accumulation of pro-IL-1β in the cytoplasm [45]. In addition, the NLRP3 inflammasome has been shown to become activated by extracellular ATP leading to caspase-1 activation and the production of functional IL-1β. There is some evidence that ATP is being sensed through the purinergic P2X7 receptor and linked to the inflammasome through pannexin-1 [46]–[48]. This therefore could explain the synergistic action of TLR signaling and ATP on IL-1β production. But what of IL-23, which is not known to require proteolytic processing? Signaling through TLRs is known to activate IL-12/23 p40 production in part through the activation of NF-kB [49]. We showed, however, that the enhancing effect of ATP was due to p19-up regulation rather than p40. Interestingly, other investigators have observed that IL-1 signaling can up-regulate IL-23 production in human DC [50] and synovialcytes [51], suggesting the possibility of an autocrine/paracrine feedback system that amplifies IL-23 by first enhancing IL-1β. There are other possibilities as well. We showed that activators of adelylate cyclase as well as cAMP could co-induce IL-23 to a similar magnitude as did ATP. In addition, adenylate cyclase antagonists partially blocked the IL-23 co-inducing action of ATP. These data suggest that the purinergic receptors of the P2Y family may play an important role in IL-23 regulation by ATP. It should be noted that P2Y11 signaling was previously shown to enhance IL-23p19mRNA [38], an observation consistent with PGE2s reported enhancement of IL-23 production since it too signals via cAMP [52] and also consistent with our observation of IL-23 regulation through the p19 subunit. It is therefore possible that the induction of the Th17-supporting DC phenotype described in this study results in part from the sensing of ATP through both P2X and P2Y families of purinergic receptors that regulate the select cytokine secretion profile through both inflammasome-dependent and independent mechanisms.

The most striking function exhibited by monocytes treated with the dual combination of ATP and LTA was the enhanced capacity to induce *in vitro* a Th17 cytokine secretion profile (IL-17^hi^/IL-22^hi^/IFN-γ^lo^), which is associated with a number of infections, chronic inflammatory pathologies and perhaps even cancer [53], [54]. This capacity appeared to be largely the result of enhanced secretion of soluble factors, since antibody neutralization of either IL-23 or IL-1β significantly suppressed IL-17 production of allo-stimulated CD4^pos^ T cells. We found that in contrast to polarized DC1, which could skew naïve Th as well as memory Th toward Th1, the capacity of ATP+LTA-treated monocytes to influence Th17 appeared confined to the memory Th compartment and not CD45RA^pos^ naïve cells. This observation is consistent with findings from several laboratories that IL-17 induction from adult peripheral T cells is often restricted to the memory subset [55], [56]. It should be noted, however, that other groups have reported success in inducing IL-17 secretion from both naïve and memory subsets [8], [57]. The reason for these divergent observations among different groups is not yet clear.

Our studies clearly show that the combination of ATP and certain TLR ligands, particularly those that signal through TLR2, can induce in monocytes a phenotype that is functionally skewed toward the support of Th17. The question remains, however, as to whether high extracellular ATP and TLR ligands actually co-exist in pathophysiological environments that are known to support chronic inflammatory reactions, particularly those containing a strong Th17 component. One interesting possibility is cancer, which has been associated (for some tumors) with chronic inflammation [58]. For example, it has been recently shown that the extracellular environment of growing tumors contains unusually high levels of ATP (in the hundred-plus micromolar range) [59], consistent with doses used in our in vitro experiments. This ATP, in conjunction with TLR agonists, could conceivably amplify select cytokines secreted by myeloid-origin cells resident in the tumor stroma, resulting in deviation of Th responses toward Th17. Such TLR agonists might be endogenous, such as the heat-shock protein gp96 or the extracellular matrix proteoglycan versican (both ligands of TLR2) [60], [61] or could even possibly come from a microbial source.

In the case of gastric cancer, for example, the microbial source of TLR agonists could be the bacterium *H. pylori*. This organism has been causally linked to a majority of stomach ulcers [62] and is now considered a serious risk factor for both gastric cancer and mucosa-associated lymphoid tissue (MALT) lymphoma [63]. Interestingly, immune responses to *H. pylori* recruit macrophages, DCs and T cells (with a strong Th17 component) to the site of infection [64], and patients with gastric carcinomas have been shown to have enhanced levels of Th17 cells in tumor-draining lymph nodes [65]. It is interesting to note that *H. pylori* LPS is also of the atypical, TLR2-activating variety [66], and this could mean that bacteria or other microorganisms with the characteristic of preferentially activating TLR 2 rather than TLR4 or other TLRs might prove especially prone to induce Th17-dominated inflammatory responses. It is therefore not difficult to conceive that many developing gastric malignancies have the potential to bring together all of the requisite cells and signaling molecules to amplify a Th17-driven immune response. It should be noted here, however, that there is not yet firm consensus regarding the precise role of Th17 immunity in promoting or controlling tumors. Instead, some groups find evidence that the IL-23/IL-17 axis favors malignancies by spurring angiogenesis, tumor growth and the tissue remodeling associated with invasion [67]–[69], while other laboratories demonstrate a potential protective role for Th17 cells [70]–[72]. It is therefore likely that the role played by Th17 cells in the promotion or control of cancer depends on many factors, including disease stage, ratio of Th17 to other Th phenotypes, and the type of malignancy in question. Despite this lack of clear consensus regarding the role of Th17 in carcinogenesis, further investigation of the role played by extracellular ATP in modulating local tumor immunity may be a fruitful avenue of exploration.

Finally, we made the interesting observation that the atypical LPS from *P. gingivalis* (that signals through TLR2) appeared greatly inferior to E. coli LPS (that signals through TLR4) for the induction of IL-12 p70 from IL-4-cultured iDC under conditions optimized for the production of that cytokine. On the other hand, the same concentrations of each LPS induced comparable levels of IL-23 when paired with ATP under IL-23-optimized culture conditions (where the cells were not exposed to IL-4 and at the time of treatment retained monocytic characteristics). This finding dovetails with a previous study done by the Vogel group [36]. They found in dose-response studies that P. gingivalis LPS was somewhat less potent than E. coli LPS for inducing common cytokines such as IL-1β and MIP-2, but higher concentrations (e.g.100 ng/ml) elicited comparable levels of these products from mouse peritoneal macrophages. Interestingly, such super-optimal concentrations of P. gingivalis and E. coli LPS nonetheless showed remarkable divergences in their respective abilities to induce other cytokines (IL-23 was not examined in these studies), suggesting possible profound biological consequences for stimulating APC with either TLR2 or TLR4 ligands. We followed the example of this study by using a super-optimal concentration of P. gingivalis and E. coli LPS (in this case 500 ng/ml) to stimulate human MoDC, thereby mitigating possible differences in LPS potency. Although sweeping conclusions cannot be drawn from these in vitro priming experiments, the differential cytokine pattern produced by DC, and the divergent abilities of these E. coli- or P. gingivalis LPS-activated APCs to promote a Th1 phenotype suggests the possibility that small differences in PAMP structure leading to differential TLR specificities can have decisive effects on APC function and subsequent Th polarization. Additional studies will be needed to assess the overall significance of this finding.

On the basis of these studies we conclude that high extracellular concentrations of ATP can influence TLR2-activated human monocytes, but not pre-differentiated monocyte-derived iDC, toward a phenotype expressing many of the characteristics of activated DC, including a specially enhanced capacity to activate Th17 responses from memory T cells. This Th17-deviating capacity appears largely a result of enhanced secretion of Th17-promoting cytokines IL-23 and IL-1β. Pathophysiological conditions that bring together high extracellular concentrations of ATP, TLR ligands, Th cells and certain myeloid-origin APC are likely to influence immune responses toward those dominated by the Th17 phenotype. The possible role of ATP in shaping the character of immune responses extant in a variety of diseases with chronic inflammatory components therefore warrants further study.
